# The Role of Tire Leachate in Condition-Specific Competition and the Persistence of a Resident Mosquito from a Competitively Superior Invader

**DOI:** 10.3390/insects13110969

**Published:** 2022-10-22

**Authors:** Oswaldo C. Villena, Joseph H. Sullivan, Edward R. Landa, Stephanie A. Yarwood, Alba Torrents, Aijun Zhang, Paul T. Leisnham

**Affiliations:** 1 Marine Estuarine & Environmental Sciences, University of Maryland, College Park, MD 20742, USA; 2 Department of Plant Science & Landscape Architecture, University of Maryland, College Park, MD 20742, USA; 3 Department of Environmental Science & Technology, University of Maryland, College Park, MD 20742, USA; 4 Department of Civil & Environmental Engineering, University of Maryland, College Park, MD 20742, USA; 5 Invasive Insect Biocontrol & Behavior Laboratory, Beltsville Agricultural Research Center-West, USDA-ARS, Beltsville, MD 20705, USA

**Keywords:** *Aedes albopictus*, *Culex pipiens*, invasive species, oviposition behavior, tire contaminants, ultraviolet radiation

## Abstract

**Simple Summary:**

Condition-specific competition is when the outcome of competition varies with environmental conditions. Tradeoffs between species’ competitive abilities and tolerances to adverse conditions are common and can facilitate or inhibit insect invasions and their impacts. Many studies have shown that the resident mosquito *Culex pipiens* persists with the competitively superior invasive mosquito *Aedes albopictus* in urban areas of the United States. Discarded vehicle tires are common developmental aquatic habitat for these species and degrade when exposed to ultraviolet (UV)-B light to release a suite of contaminants. We tested the hypothesis that more highly degraded tires that contain greater amounts of contaminants produce a condition-specific advantage for *Cx. pipiens* by altering the outcome of competition with *Ae. albopictus*. We found stronger competitive effects of *Cx. pipiens* on the population performance and survival of *Ae. albopictus* in tires exposed to shade and full-sun conditions that had higher concentrations of tire leachate than no UV-B conditions. This suggests that increased tire degradation and tire leachate promotes condition-specific competition and facilitates the regional persistence of *Cx. pipiens* after the invasion of *Ae. albopictus.*

**Abstract:**

(1) Background: Condition-specific competition, when the outcome of competition varies with abiotic conditions, can facilitate species coexistence in spatially or temporally variable environments. Discarded vehicle tires degrade to leach contaminants into collected rainwater that provide habitats for competing mosquito species. We tested the hypothesis that more highly degraded tires that contain greater tire leachate alters interspecific mosquito competition to produce a condition-specific advantage for the resident, *Culex pipiens*, by altering the outcome of competition with the competitively superior invasive *Aedes albopictus*. (2) Methods: In a competition trial, varying densities of newly hatched *Ae. albopictus* and *Cx. pipiens* larvae were added to tires that had been exposed to three different ultraviolet (UV)-B conditions that mimicked full-sun, shade, or no UV-B conditions in the field. We also measured *Cx. pipiens* and *Ae. albopictus* oviposition preference among four treatments with varying tire leachate (high and low) and resources (high and low) amounts to determine if adult gravid females avoided habitats with higher tire leachate. (3) Results: We found stronger competitive effects of *Cx. pipiens* on the population performance and survival of *Ae. albopictus* in tires exposed to shade and full-sun conditions that had higher concentrations of contaminants. Further, zinc concentration was higher in emergent adults of *Ae. albopictus* than *Cx. pipiens*. Oviposition by these species was similar between tire leachate treatments but not by resource amount. (4) Conclusions: These results suggest that degraded tires with higher tire leachate may promote condition-specific competition by reducing the competitive advantage of invasive *Ae. albopictus* over resident *Cx. pipiens* and, combined with *Cx. pipiens’* preferential oviposition in higher resource sites, contribute to the persistence of the resident species.

## 1. Introduction

Understanding ecological mechanisms that govern the establishment and spread of exotic insects and their impacts on resident communities is of fundamental importance to the field of invasion biology. Traditional niche theory and empirical research indicate that superior competitive ability can determine the success and impacts of exotic species [1,2,3]. With one limiting resource in a constant environment, interspecific competition should result in competitive exclusion [4,5], and there are numerous instances where a competitively superior invader has displaced resident species’ [6,7]. However, there is also evidence of competitively inferior residents escaping competitive exclusion via a number of mechanisms, including differential resource use [4,8], habitat segregation [9,10], and trade-offs between competitive ability and environmental tolerances [11,12]. Condition-specific competition, whereby abiotic conditions reduce or reverse the outcome of competition between two species, can facilitate coexistence when there are temporal or spatial changes in conditions [13,14,15]. Condition-specific competition is perhaps most interesting and important when it facilitates the persistence of a resident species despite the spread of a competitively superior invader, resulting in altered ecological, economic, and human health impacts.

The U.S. Tire Manufacturers Association estimates that around 303.5 million automobile tires are used annually in the United States [16]. When tires degrade, a suite of organic (e.g., polycyclic aromatic hydrocarbons, benzothiazoles, 4-tert-butylphenol) and inorganic (principally zinc, a process reagent in the manufacture of tread material, [17]) contaminants collectively known as ‘tire leachate’ can filter into aquatic environments [18,19,20]. Tire degradation and resultant tire leachate is influenced by a number of environmental factors, including temperature, ozone, and humidity, but arguably the most important factor is exposure to ultraviolet (UV) radiation [21,22]. UV radiation with a wavelength between 315 and 280 nm, classified as UV-B radiation, is the more damaging to tire structure because of its shorter wavelength and higher energy levels compared with other forms of UV radiation [21].

Tire leachate and some of its individual constituent compounds have been documented to negatively affect a range of aquatic biota, including fish [18,23], amphibian embryos [24,25], algae [25], bacteria [18,23,26], planktonic crustaceans [27,28,29,30], and insects [31]. All of these studies focused on the direct effects of tire leachate or individual compounds on the survival of a single target species [18,32]. Yet, the most interesting and important effects from tire leachate may instead be the alteration of species interactions that structure ecological communities. There is increasing empirical evidence that environmental contaminants often have community-level impacts [33]. For example, some contaminants have been shown to negatively affect organisms’ susceptibility to predation [34,35,36], susceptibility to disease [37], and ability to gather resources [38], as well as lower the availability of resources [34], impair reproduction [36], and increase the bioaccumulation and biomagnification of other contaminants [39,40,41]. Effects of contaminants can appear to be lethal and expressed through reduced survival, or non-lethal and expressed through reduced growth and longer development times [25,34]. Furthermore, both lethal and non-lethal effects acting at one trophic level (e.g., resources) likely have impacts on other trophic levels (e.g., competitors). The complex composition of these effects is likely to variably influence species interactions and distributions as they vary across the environment [33,42,43]. It is possible for tire leachate to interfere in the outcome of interspecific competition if one species is more exposed or susceptible to its constituent contaminants. This type of condition-specific competition may impact the success and impacts of biological invasions yet has not been studied.

Container-dwelling mosquitoes that utilize discarded tires provide a convenient model to investigate the role of condition-specific competition in biological invasions and their impacts on resident species. Discarded tires collect rainwater and provide large developmental habitat for many mosquitoes [44]. Tires have been particularly instrumental in the establishment and spread of invasive mosquitoes [45,46]. The best documented mosquito invasion is that of *Aedes albopictus* (Skuse 1894) in North America, which is thought to have arrived from Asia through tire shipments in the 1980s [46,47]. In the Mid-Atlantic region of the United States, *Ae. albopictus* often co-occurs in tire habitats with the resident species, *Culex pipiens* (Linneaus 1758), where larvae of both species compete for limited microbial food resources associated with allochthonous inputs of plant and animal detritus [44,48]. Three laboratory studies have tested competition between North American strains of *Ae. albopictus* and *Cx. pipiens*, and all convincingly demonstrate overwhelming competitive superiority for *Ae. albopictus* under almost all conditions [15,49,50], which is consistent with studies in Europe [51,52], but see also [53]

Despite being competitively inferior, *Cx. pipiens* has persisted in urban areas where tires are common after the invasion of *Ae. albopictus*, and is frequently collected from the same individual tire habitats with the invasive species [31,50]. Differential life history traits of *Ae. albopictus* and *Cx. pipiens* support the idea that tire leachate might modify the outcome of interspecific competition between these two species. *Cx. pipiens* is well-documented to utilize habitats with high concentrations of nutrients and organic and inorganic pollutants when they are available (e.g., septic tanks) suggesting that it is more tolerant to environmental contaminants than *Ae. albopictus* which tends to be restricted to less polluted habitats [48,54]. Consistent with this idea, empirical studies show that *Ae. albopictus* has shown reduced performance in habitats with excessive nutrient pollutants [55]. Furthermore, *Cx. pipiens* and *Ae. albopictus* have different feeding behaviors. *Aedes albopictus* spends much more time feeding than *Cx. pipiens* and a greater proportion of its feeding time browsing surface biofilm compared with *Cx. pipiens* which tends to exclusively filter-feed in the water column [56]. These different feeding patterns suggest that *Ae. albopictus* might be more exposed to tire contaminants that leach from the tire wall and concentrate in surface biofilms.

There are increasing literature illustrating the importance of condition-specific competition in structuring ecological communities, including the invasion and impacts of *Ae. albopictus.* For example, Alto et al. [57] found that field concentrations of malathion, an organophosphate insecticide, was able to eliminate competitive superiority of *Ae. albopictus* over *Ae. aegypti*. Costanzo, Kesavaraju and Juliano [9] demonstrated a reversal of competitive superiority in favor of *Ae. aegypti* over *Ae. albopictus* in dry conditions due to greater egg mortality, and Müller, Knautz, Vollroth, Berger, Kreb, Reuss, Groneberg and Kuch [53] showed greater larval survival of *Cx. pipiens* than *Ae. albopictus* at lower temperatures. Last, Leisnham, LaDeau, Saunders and Villena [50] found increased densities of *Ae. albopictus* negatively affected the survivorship and development of *Cx. pipiens* in water from discarded, but not functional, containers, driven mainly by water from trash cans that had which allowed consistently higher *Cx. pipiens*’ survival and development and had increased nutrient concentrations.

Collectively, *Ae. albopictus* and *Cx. pipiens* are vectors for a range of human and animal pathogens, including West Nile virus (WNV), dengue, yellow fever, Eastern Equine encephalitis, La Crosse encephalitis, St. Louis encephalitis, Japanese encephalitis, avian malaria, and dog heartworm [58,59,60], thus the distribution and abundance of each species is of medical and veterinary importance. Coexistence of *Ae. albopictus* and *Cx. pipiens* may be particularly important for the spread of human WNV in the United States and other regions where they are found, including Europe and South Africa [61]. *Culex pipiens* is the main WNV vector of avian species that serve to amplify the virus in urban areas [60], and often bite mammals to bridge WNV into human populations [60,62]. The persistence of *Cx. pipiens* after *Ae. albopictus* invasion where WNV is present is likely to maintain existing enzoonotic circulation and human transmission of the virus. *Ae. albopictus* is likely to also act as an additional bridge of WNV into human populations [63,64]. Thus, the coexistence of *Cx. pipiens* and *Ae. albopictus* is likely to increase WNV transmission if they contribute to simultaneous zoonotic and bridge transmission.

The aim of this paper was to test the effect of tire degradation from UV-B radiation on interspecific competition between *Ae. albopictus* and *Cx. pipiens*. We subjected replicate vehicular tires to three different levels of UV-B exposure that mimicked full sun, shade, or no UV-B conditions in the field in a controlled greenhouse experiment. We tested the hypothesis that more highly degraded tires that contain greater tire leachate alters the outcome of interspecific mosquito competition, producing a condition-specific advantage for the competitively inferior resident, *Cx. pipiens*, by relaxing the effects of competition with the invasive *Ae. albopictus*. From this hypothesis, we drew the following predictions: 1. Tires exposed to greater UV-B radiation representative of full sun conditions will have higher concentrations of tire leachate than tires exposed to shade conditions or no UV-B conditions; and 2. *Cx. pipiens* will be more competitive versus *Ae. albopictus* in tires exposed to higher UV-B radiation that have greater tire leachate. For tire leachate to facilitate the coexistence of *Ae. albopictus* and *Cx. pipiens* through the mechanism of condition-specific competition, both species need to utilize the same tire habitats so that larval competition for food resources has the potential to occur. An alternative but not mutually exclusive hypothesis of coexistence between *Ae. albopictus* and *Cx. pipiens* is habitat segregation among tires that vary in leachate concentration. This hypothesis appears unlikely since prior studies have commonly collected both species together in the same individual tires [44,48,50]. Nevertheless, we tested the effect of tire leachate and the amount of food resources on the oviposition preference of gravid female *Ae. albopictus* and *Cx pipiens* in a laboratory oviposition choice trial.

## 2. Materials and Methods

### 2.1. Tire Condition Experiment

To assess the effects of UV-B radiation on the release of tire leachate, used tires were exposed to varying UV-B radiation conditions in 6 replicate blocks of 15 tires each using a repeated measure randomized complete block design (RCBD). The experiment was conducted in a controlled greenhouse facility at the University of Maryland that was regulated at 25 °C, >80% RH, and 16:8 L:D h photoperiod. Polyester filters that block almost all UV-B radiation below 316 nm were applied to the windows of the greenhouse facility to exclude outside UV-B radiation. Five tires within each block were randomly assigned to one of three benches that were exposed to one of three common conditions determined by field measures: (1) full-sun: 10.82 μmol/m^2^/s (FS); (2) shade: 6.1 μmol/m^2^/s (S); and (3) no UV-B radiation: 0.6 μmol/m^2^/s (NUV). UV-B radiation was provided by 12 UVB-313 lamps (Q Panel Lab Products, Cleveland, OH, USA). UV-B lamps for FS and S conditions were wrapped with cellulose diacetate (CA) biofilm, which transmitted UV-B radiation down to 290 nm, while. UV-B lamps for the NUV condition were wrapped in polyester filters [65,66]. Full-sun and shade condition lamps were adjusted to a height of 0.6 and 1.2 m above tires to attain the appropriate UV-B radiation levels, which were confirmed for all treatment conditions using an UV meter (UVM-SS, Apogee Instruments Inc., Logan, UT, USA) [66]. To assure uniform exposure to UV-B radiation, tires in each block were rotated in their fixed position on the bench every 4 days, and each block was run for 150 days in sequential temporal order. 

Tires were filled with 4 L deionized (DI) water, which was maintained by routine additions throughout the duration of the experiment [67]. Water samples were taken from each tire on days 1, 50, 100, and 150 after tire set-up, and they were acid digested following the U.S. EPA 3015A method [68]. Analysis of digested water samples for total and dissolved zinc concentrations were conducted with an Inductively Coupled Plasma Atomic Emission Spectroscopy (ICP-AES) with autosampler following the U.S. EPA Method 200.7 [69]. Standardization of equipment occurred after daily calibration and after every 11 samples. Calibration standards were 0, 0.4, 2, 4, and 6 mg zinc/L in 5% (v/v) nitric acid. Standardization regression was linear up to 100 mg zinc/L. Samples were not taken from tires on day 100 in block 1 and seven samples across all blocks were excluded from analysis because of contamination, resulting in 62 total water samples (Dataset S1). 

All tires were the same brand and type (Goodyear, model Assurance: P215/60R16) and sourced from the University of Maryland Motor Transportation Services who replace tires after 30,000 miles of wear, ensuring that the tires in this study had been exposed to similar conditions before being used in the experiment. 

### 2.2. Competition Trial

After tires in blocks 3-6 had been exposed to UV-B radiation conditions for 150 days, they were entered in the mosquito competition trial using a split-plot randomized complete block design, with UV-B radiation condition as the main plot and competition treatment as the sub-plot. Because the first block of tires was solely used to assess effects of UV-B radiation and because there were insufficient numbers of larvae for one of the five other blocks, four replicate blocks of tires (60 total) were used for the competition trial. For each block, newly hatched *Ae. albopictus* and *Cx. pipiens* larvae were added in varying densities. Each of the five tires within each of the three UV-B radiation conditions were randomly assigned one of five mosquito competition treatments (*Ae. albopictus*: *Cx. pipiens*, 0:100, 0:50, 100:0, 50:0, 50:50) and provisioned with 1.0 g senescent dried white oak (*Quercus alba*) leaf litter a week earlier to create a response-surface competition trial. Treatments with 100 single species larvae (i.e., 0:100, 100:0) and with 100 mixed species larvae (i.e., 50:50) were expected to exert high competition compared with treatments with only 50 single-species larvae (e.g., 0:50, 50:0), which would exert little competitive pressure and represent “baseline” conditions. These per capita resource amounts (0.01–0.02 g per larva) are similar to those of prior studies testing competition between mosquitoes in tires [70,71,72]. Before the addition of larvae, UV-B lamps were wrapped with polyester filters to prevent UV-B radiation from directly affecting larvae or their microbial food, yet still remained on retain the 16:8 L:D h photoperiod [73].

After larvae were added for each block, tires were checked daily to collect pupae, which were then placed in individual vials with water until adult emergence. Upon emergence, adults were killed by drying them at >40 °C for 48 h. From each tire, three fitness parameters were measured for both species: proportion female survival, median female development time, and median female wing length as a measure of body size. These fitness parameters were used to estimate the finite rate of population increase (*λ*′) [73]:

$$\lambda^{\prime }=\exp\left[\frac{\log\left[\left(1/N_{0}\right)\underset{x}{\sum }A_{x}f\left(w_{x}\right)\right]}{D+\left[\underset{x}{\sum }xA_{x}f\left(w_{x}\right)/\underset{x}{\sum }A_{x}f\left(w_{x}\right)\right]}\right]$$

where *N*_0_ is the initial number of females per container or microcosm (assumed to be 50% of the larvae population); *Ax* is the number of females eclosing on day *x*; *wx* is a measure of median female size on day *x*; *f(wx*) is a function relating fecundity to female size, and *D* is the estimated time (in days) required for a newly eclosed female to mate, obtain a bloodmeal, and oviposit. *D* is assumed to be 10 days for *Cx. pipiens* [15,74], and 14 days for *Ae. albopictus* [73]. For *Ae. albopictus*, *f(wx)* = 78.02*wx*-121.24 (r^2^ = 0.713, N = 91, *p* < 0.0001) [75] and for *Cx. pipiens*, *f(wx)* = 148.5*wx* − 383.82 (r^2^ = 0.3724, N = 55, *p* < 0.0001) [76] (Dataset S2).

At the end of the mosquito competition trial for blocks 5 and 6, each tire was destructively sampled and all contents (biofilm, detritus, remaining larvae, water) were separated and removed. Biofilm, the assemblage of surface-associated microbial material along tire walls, was harvested and dried for 24 h at 95 °C and then at 105 °C for 4 h to obtain its total dry weight (Dataset S3). A 0.3 g dried sample of biofilm from each tire was analyzed for total zinc using an Inductively Couple Plasma Optical Emission Spectroscopy (ICP-OES) (PerkinElmer Inc.; Waltham, MA, USA). One dried biofilm sample was too small to be analyzed for total zinc, resulting in 29 samples (Dataset S3). Dried and measured adults from tires in blocks 3 and 4 were also tested for total zinc, after being pooled by UV-B radiation condition and species to yield sufficient material (12 total samples). As with water samples (described above), biofilm and mosquito samples were acid digested following the U.S. EPA 3015A method [68] and measured for total zinc using an ICP-OES following the U.S. EPA Method 200.7 [69] (Dataset S4).

### 2.3. Oviposition Choice Trial

An oviposition choice trial was conducted to assess the effects of tire leachate (high and low zinc concentration) and resources (high and low microbial concentrations) on oviposition preference using a two-factor RCBD. After block 6 of the competition trial (day 210), water was collected from tires with the highest and lowest tire leachate (dissolved and total zinc: 4.11 and 6.24 mg/L vs. 0.18 and 0.20 mg/L) to yield two different tire leachate treatment levels. Half the water from each treatment level was then filtered (0.22 µm pore-size, Corning, Glendale, CA, USA) to remove microorganisms and yield a low resource treatment level compared to unfiltered water that represented a high-resource treatment level. One 1-mL sample from each of the four leachate-resource treatment combinations was used to measure microbial activity as the rate of heat production (µwatts/mL) using a multicell differential scanning calorimeter (MC-DSC model 4100, Calorimetry Sciences Corp., Lindon, UT, USA) in isothermal mode at 25 °C ± 0.05 following procedures of past studies [73,77,78]. Resource treatment levels showed a statistically significant difference in microbial metabolic rates (F_1,3_ = 661.8, *p* < 0.0001), with lower metabolic rates for filtered tire leachate samples (Filtered high zinc: 0.279 ± 0.077 µwatts/mL, Filtered low zinc: 0.341 ± 0.091 µwatts/mL; Unfiltered high zinc: 5.840 ± 0.245 µwatts/mL, Unfiltered low zinc: 6.295 ± 0.355 µwatts/mL). No main effect of zinc concentration (F_1,3_ = 1.38, *p* = 0.247) or interaction effect of zinc concentration and filtering on microbial activity were detected (F_1,44_ = 0.74, *p* = 0.395).

Twenty (20) female *Ae. albopictus* or *Cx. pipiens* were released into experimental cages (30 cm^3^) holding four oviposition cups (200 mL black plastic cups lined with a paper towel for *Ae. albopictus* or with 0.1 g of foxtail grass, previously rinsed with sterile water, for *Cx. pipiens*) positioned in opposite corners and randomly assigned one of the four tire leachate-resource treatment combinations. Two blocks of six cages (three cages per species) were housed in each of two identical environmental chambers (Model I-36 VL; Percival Scientific Inc., Perry, IA, USA) set at 25 °C, >80% RH, and at 16:8 L:D h photoperiod. All females had been recently (<24 h) blood-fed to repletion. Each cohort of 20 females were released with 5 males of the same species to ensure the production of fertile eggs. Females were allowed to oviposit for 7 days and had continuous access to sugar water (20% v/v) to facilitate their survival. For *Ae. albopictus*, papers were removed on day 7 and the number of individual eggs were counted per cup in each of its six cages using a magnification stereo microscope with a 10X dual magnification (Fisher Scientific, Pittsburgh, OH, USA), resulting in 24 total measures of oviposition (Dataset S5). For *Cx. pipiens*, the number of egg rafts laid per cup were recorded in each of its six cages for a second set of 24 oviposition measures (Dataset S6). Oviposition-mediated habitat segregation between *Ae. albopictus* and *Cx. pipiens* larvae could be due to two main processes: different oviposition preference, whereby gravid females choose different developmental habitat to oviposit eggs, or differential egg survival [79]. In this study, we only focused on comparing oviposition preference considering that egg survival depends on many environmental conditions (e.g., temperature, precipitation) and is not the focus of this oviposition trial. Furthermore, some evidence suggests that tire leachate is unlikely to affect egg survival of either species [80]. Although, female mosquito oviposition can be influenced by other females [80], we chose to use cohorts of 20 females to reduce data stochasticity and because cohorts are more realistic of field conditions where multiple females are likely to oviposit in tire habitats.

All mosquitoes in both the competition and oviposition trials were F_1-3_ generation individuals from colonies housed in an insectary at 25 °C, >80% RH, and at 16:8 L:D h photoperiod. Colonies had been established from multiple collections of larvae in tires and other container habitats in the Washington, District of Columbia-Baltimore, Maryland metropolitan area. Neither *Ae. albopictus* or *Cx. pipiens* are endangered and collection sites were either on publicly accessible lands or on private lands where consent was granted at the time of collection; thus, no field permits were required to collect them. Field-collected larvae were raised to adulthood on lactalbumin (MP Biomedicals LLC, Solon, OH, USA) and adult females were fed horse or rooster blood via an artificial feeder (Hemotek, Accrington, UK) to produce eggs.

### 2.4. Statistical Analyses

ANOVAs were used to test effects of UV-B radiation and other predictors on response variables using SAS Proc Mixed [81] with experiment-wise α = 0.05. A first set of ANOVAs were used to test effects of UV-B radiation condition on total and dissolved zinc concentrations in tire water (mg/L), biofilm amount per tire (g), and total zinc concentration in biofilm (mg/g). Models of zinc concentrations in water were separated by sample day to meet the assumptions of normality and homogeneity of variances and included measurements at day 1 (study baseline) as a covariate. Replicate block was included as a random effect and experimental units were groups of 5 tires under the same UV-B radiation condition per block, with each individual tire treated as a sub-sample. Models of biofilm dry weight and total biofilm zinc concentration included UV-B condition as a fixed effect. Replicate blocks and tires nested in replicate blocks were included as random effects. Differences among UV-B radiation conditions were determined using post hoc pairwise tests with Tukey adjustment.

A second set of ANOVAs were used to test the effects of UV-B radiation condition and competition treatment on Cx. pipiens and Ae. albopictus per capita rate of population change (*λ*′), survival, median female development time, and median female wing length. A significant interaction between UV-B radiation condition and competition treatment would indicate that UV-B radiation condition altered the effect of competition. Pairwise contrasts were used to determine differences among UV-B conditions within competition treatment and differences among competition treatments within UV-B condition [82], with sequential Bonferroni correction for all possible comparisons (9) within each family of analyses. In all models, replicate block was included as a random effect. To account for assumptions of normality and homogeneity of variances, all survival data were arcsin transformed and *Ae. albopictus λ*′ was log10(y+1) transformed. Despite transformations, *Cx. pipiens λ*′ failed to meet parametric assumptions, thus a randomization ANOVA was used (Randomization wrapper for SAS PROCs; Cassell 2011). Randomization ANOVA yielded conclusions identical to those of parametric ANOVA, suggesting that ANOVA results were minimally influenced by departures from normality or homogenous variance. For brevity, only the results from parametric ANOVA are presented. A follow-up ANOVA was used to test for effects of UV-B condition, species, and their interaction on zinc concentration in emerged adults. To account for assumptions of normality and homogeneity of variances, mosquito zinc concentration was log10(y+1) transformed and replicate block was included in the model as a random effect. Differences among UV-B conditions were determined using post hoc pairwise tests with Tukey adjustment.

A third set of ANOVAs were used to test the effects of UV-B radiation condition and resources on numbers of *Ae. albopictus* eggs and numbers of *Cx. pipiens* egg rafts. In all models, tire leachate concentration and resources were included as fixed effects, incubators (blocks) were included as random effects, and differences in tire leachate concentration and resources were determined using post hoc pairwise tests with Tukey adjustment.

## 3. Results

### 3.1. Tire Condition Experiment

**Figure 1:**
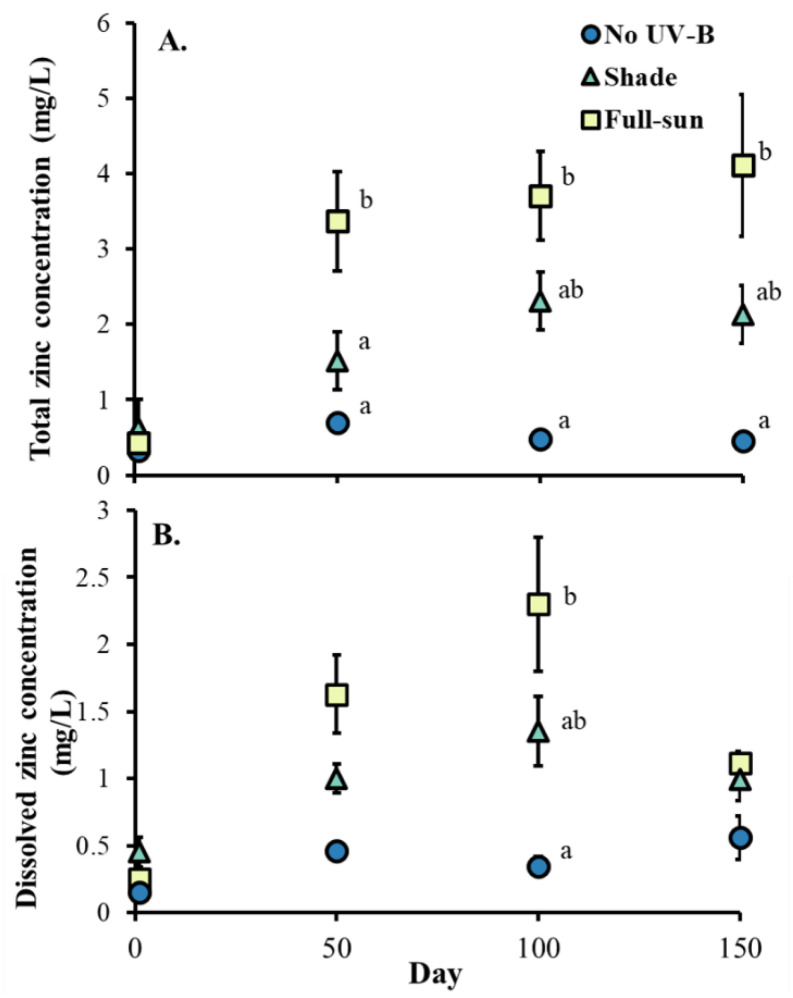
Least square means (±SE) for (**A**) total and (**B**) dissolved zinc concentration in water from tires exposed to UV-B radiation conditions that mimicked full-sun, shade, and no UV-B radiation conditions on days 50, 100, 150. Significant pairwise comparisons within each day are indicated by different letters beside markers (*p* < 0.05). No letters indicate no significant pairwise differences.

UV-B radiation affected total zinc concentration in tire water, showing significant differences among conditions at days 50 (F_2,11_ = 7.21, *p*-values = 0.010), 100 (F_2,11_ = 11.81, *p* -values = 0.004), and 150 (F_2,11_ = 4.24, *p*-values = 0.045). Dissolved zinc concentration was also affected by UV-B radiation at day 100 (F_2,11_ = 4.50, *p*-values = 0.047), but not at days 50 (F_2,11_ = 3.75, *p*-values = 0.057) or 150 (F_2,11_ = 2.54, *p*-values = 0.117). In all instances, zinc concentration was higher in tires exposed to conditions mimicking full sun than no UV-B conditions, and at day 50, total zinc concentration was also higher in tires exposed to conditions mimicking full sun than tires exposed to conditions mimicking shade (Figure 1A, B).

**Figure 2:**
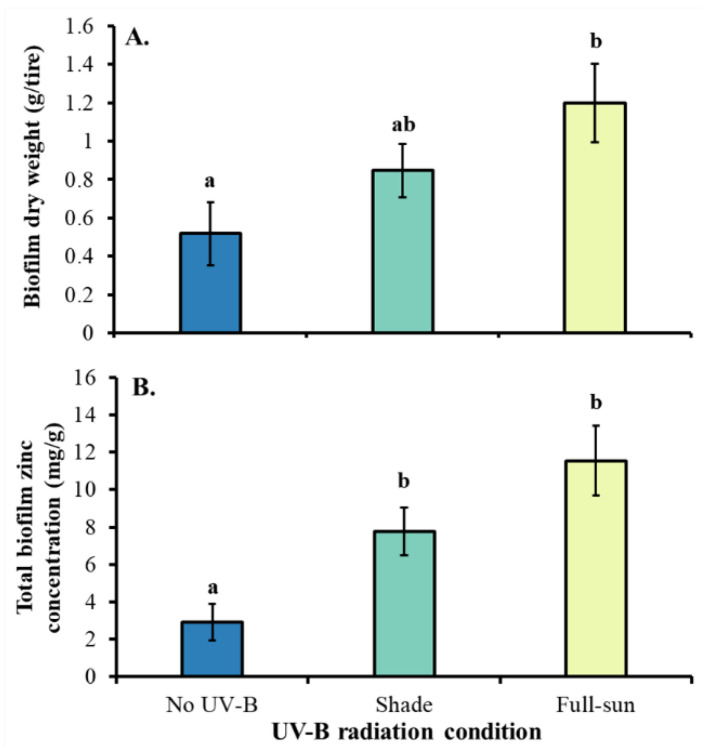
Least square means (±SE) for (**A**) dry weight of tire biofilm (g per tire) and (**B**) total zinc concentrations in biofilm (mg/g) from tires exposed to UV-B radiation that mimicked no UV-B, shade, or full-sun conditions. Data were statistically tested using ANOVA. Significant pairwise comparisons are indicated by different letters above bars (*p* < 0.05).

UV-B radiation also affected biofilm mass (F_2,25_ = 7.95, *p* = 0.002), with higher amounts under full-sun conditions than under no UV-B conditions (Figure 2A), and zinc concentrations within biofilm (F_2,25_ = 22.13, *p* < 0.001), with higher total zinc concentrations in biofilm from tires exposed to shade and full-sun conditions compared to tires exposed to no UV-B conditions (Figure 2B).

### 3.2. Competition Trial

**Figure 3:**
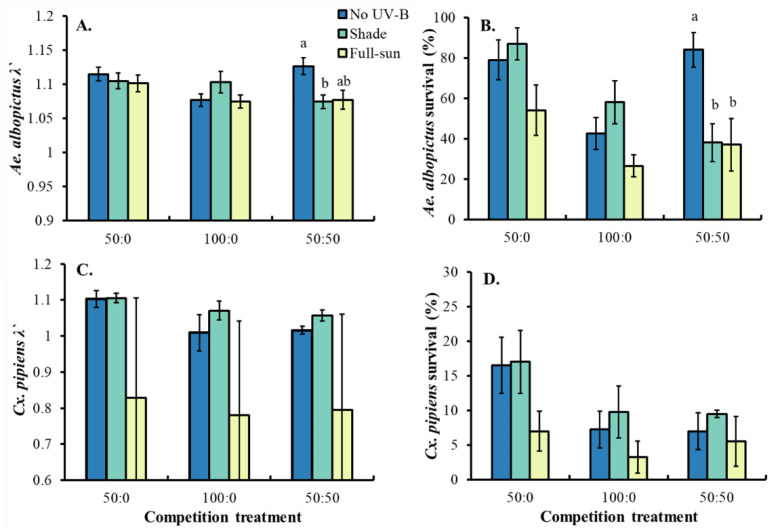
Least square means (±SE) of the interaction effects of UV-B radiation (no UV-B, shade, full-sun) and competition treatment (50:0, 100:0, 50:50; conspecifics:heterospecifics) on the estimated finite rate of population increase (λ’) and survival (%) of (**A**,**B**) *Ae. albopictus* and (**C**,**D**) *Cx. pipiens*. Data were statistically tested using ANOVA. Significant pairwise comparisons among treatment levels are indicated by different letters above bars (*p* < 0.05). No letters indicate no significance.

Across all UV-B radiation conditions and competition treatments, *Ae. albopictus* had higher *λ*′ than *Cx. pipiens* (Figure 3), indicating that it was the superior competitor. Nevertheless, there was a significant interaction of UV-B radiation condition and competition treatment on both *Ae. albopictus λ*′ and survival (Table 1). *Ae. albopictus λ*′ and survival were lower in tires exposed to S conditions compared to NUV conditions under *Cx. pipiens* (50:50) but not conspecific (50:0, 100:0) competition (Figure 3A,B), indicating that UV-B radiation of tire habitats altered the response of *Ae. albopictus* to *Cx. pipiens* competition. *Ae. albopictus* survival was also lower in tires exposed to FS conditions compared to NUV conditions (Figure 3B), while *λ*′ showed a similar but not significant trend (Figure 3A). Main effects of UV-B condition and competition were detected on Ae. albopictus survival (Table 1), with lower survival in tires exposed to FS conditions compared to S and NUV conditions (Figure S1), and with higher conspecific (100:0) and Cx. pipiens (50:50) competition (Figure S1). Aedes albopictus female development time and female wing length were not affected either by UV-B radiation condition or competition treatment (Table 1). For Cx. pipiens, there was no interaction between UV-B condition and competition treatment for *λ*′ or its individual fitness parameters (Table 1, Figure 3C,D), indicating no evidence that tire exposure to UV-B radiation altered the impact of competition on Cx. pipiens performance (Figure 3). Nevertheless, there were main effects of UV-B condition on Cx. pipiens survival and female wing length (Table 1). There was higher survival in tires exposed to S and NUV conditions than FS conditions (Figure S1), while surviving larvae in S and FS conditions developed into larger adults than larvae in tires exposed to NUV conditions (Figure S2). Main effects of competition treatments were also detected on Cx. pipiens survival, female development time, and female wing length (Table 1). Culex pipiens survival was lower and female wing length shorter under higher competition from Ae. albopictus (50:50) and conspecifics (100:0) (Figure S2), while female development time was longer under Ae. albopictus competition (50:50) compared to low conspecific competition (0:50) (Figure S2).

**Figure 4:**
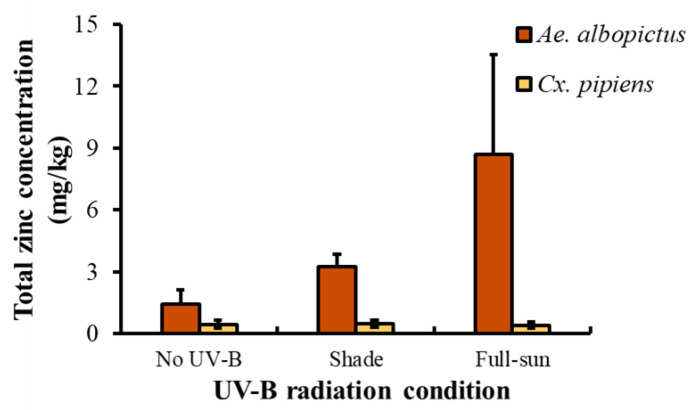
Least square means (±SE) total zinc concentration in mosquitoes (mg/kg) for *Ae. albopictus* and *Cx. pipiens* in response to UV-B radiation treatment. Data were statistically tested using ANOVA. No letters indicate no significant pairwise differences (*p* > 0.05).

There was a main effect of species on total zinc concentration (F_1,5_ = 29.44, *p* = 0.0029), with *Ae. albopictus* having greater zinc concentration than *Cx. pipiens* across all UV-B conditions. Zinc concentration trended higher across UV-B radiation conditions, from NUV through S to FS (Figure 4), but this relationship was not significant (F_2,5_ = 1.67, *p* = 0.2775) nor was the species x UV-B condition interaction (F_2,5_ = 2.19, *p* = 0.2072).

### 3.3. Oviposition Choice Trial

**Figure 5:**
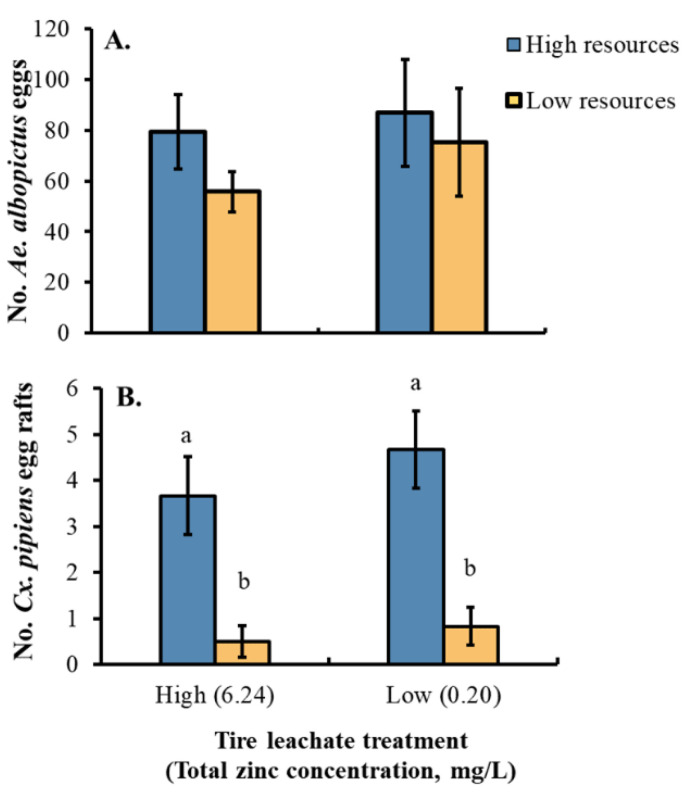
Least squares means (±SE) for (**A**) numbers of oviposited *Ae. albopictus* eggs and (**B**) numbers of *Cx. pipiens* egg rafts by tire leachate concentration (high, low) and food resources (high, low). Data were statistically tested using ANOVA. Significant pairwise comparisons among treatment levels are indicated by different letters above bars (*p* < 0.05). No letters indicate no significant pairwise differences.

There were no main or interaction effects of leachate concentration or resources on *Ae. albopictus* oviposition (F-values_1,3_ = 0.12–1.07, *p*-values = 0.444–0.734; Figure 5A). There was no effect of leachate concentration on *Cx. pipiens* oviposition but a main effect of resources was detected, with 4–5 times the number of egg rafts oviposited in cups with high resources than those with low resources across the two zinc concentration treatments (F_1,3_ = 29.87, *p* < 0.05; Figure 5B; interaction effect: F_1,3_ = 0.615).

## 4. Discussion

Condition-specific competition, when the outcome of competition varies with environmental conditions, can influence the composition and structure of insect communities [9,83,84,85]. Our study suggests condition-specific competition, through the moderating effects of tire degradation from UV-B radiation and resultant exposure to tire contaminants, alters the outcome of competition between invasive *Ae. albopictus* and native *Cx. pipiens.* Our prediction of increased *Cx. pipiens* competitiveness versus *Ae. albopictus* in habitats with higher tire leachate was supported. Our results show lower *Ae. albopictus* per capita rate of population increase (*λ*′) in response to *Cx. pipiens* competition in tires exposed to higher UV-B-radiation and with higher zinc concentrations, a common marker of tire leachate. Prior studies have documented the effects of UV-B radiation on tire degradation [22,86], and negative effects of tire leachate or its individual constituent compounds on aquatic biota [23,26,27,31], but this is the first study to illustrate that negative effects of tire leachate from realistic UV-B conditions can alter species interactions and the impacts of a biological invasion.

We found no evidence that tire leachate concentration altered the oviposition of either *Ae. albopictus* or *Cx. pipiens,* but that *Cx. pipiens* preferred to oviposit in cups with higher resources. *Cx. pipiens’* preference for experimental cups with higher resources is broadly consistent with several studies showing that *Cx. pipiens* preferentially oviposit in habitats with high organic content [87,88]. Distributions of *Ae. albopictus* and *Cx. pipiens* are probably affected by several environmental variables acting at different scales, including container resources, size, and temperature, adult resting sites, and host densities. Yet, numerous field surveys have collected *Ae. albopictus* and *Cx. pipiens* co-occurring in the same individual tires [44,48,50]. Our findings suggest that condition-specific competition in resource-limited tires, as well as the relaxation of competition in resource-rich habitats, are both plausible mechanisms contributing to the regional persistence of *Cx. pipiens* in response the invasion of the competitively superior *Ae. albopictus.* This study adds to growing literature demonstrating the importance of condition-specific competition on the dynamics of ecological communities and the invasion and impacts of *Ae. albopictus* in particular [9,50,53,57].

Although *Ae. albopictus* was competitively superior to *Cx. pipiens* across all UV-B conditions and density treatments, there was little evidence that tire degradation altered the effects of *Ae. albopictus* competition on *Cx. pipiens.* Like *Ae. albopictus*, survival of *Cx. pipiens* was on average lowest in tires exposed to the FS condition, which had the greatest UV-B radiation and had the highest mean concentration of tire leachate. However, unlike *Ae. albopictus,* whose body size and development time did not vary across density treatments or radiation conditions, *Cx. pipiens* survivors developed faster and were larger in tires that had been exposed to higher UV-B radiation, resulting in no difference in *Cx. pipiens λ*′ with UV-B condition. A species’ competitive ability results from several traits that constitute its effects on other species and its response to interspecific competition. Competitive effect is usually associated with the ability to harvest and deplete scarce resources [4]. Harvesting efficiency can contribute to competitive response, but response is also affected by physiological efficiency and flexibility, such as reduced metabolic demands or plasticity of size and time to maturity that can enable a species to maintain high population growth despite competition [4]. It is likely that variation in both competitive effects and responses resulted in the observed differences among UV-B radiation conditions in our study. *Cx. pipiens* survival was similar in tires exposed to NUV and S conditions, but it is likely that the larger *Cx. pipiens* larvae in the S condition depleted more microbial resources and exerted a greater competitive effect than the smaller larvae in the NUV condition, causing significantly lower *Ae. albopictus λ*′. *Cx. pipiens* larvae were similarly larger in the FS condition but their lower survival caused insufficient competitive effect on *Ae. albopictus* to result in a significant difference in *Ae. albopictus λ*′ between FS and NUV conditions.

Lower *Ae. albopictus* performance in response to *Cx. pipiens* competition in both the FS and S conditions may also have been because of increased metabolic demands and reduced developmental plasticity when resisting tire contaminants. Adult *Ae. albopictus* emerged with higher zinc concentrations than *Cx. pipiens* across all radiation conditions. *Ae. albopictus* also showed increasing zinc concentrations from NUV to FS conditions, although this trend was not statistically significant presumably because of low sample sizes. *Ae. albopictus* spends more time feeding and a greater proportion of its feeding time browsing surface biofilm than *Cx. pipiens* [56], which probably exposes it to greater concentrations of leachate from tire surfaces. It is possible that increased competition from larger *Cx. pipiens* larvae, combined with degraded physiological efficiency and flexibility from higher exposure to tire contaminants, resulted in *Ae. albopictus* having lower performance when it was co-occurring with *Cx. pipiens* in more degraded tires. Although we observed lower survival of both *Ae. albopictus* and *Cx. pipiens* in higher UV-B radiation conditions (FS, S) and in higher competition treatments (100:0, 50:50), we saw no main effects of UV-B radiation or competition treatment on *λ*′ of either species. If *Ae. albopictus’* greater exposure to tire contaminants only caused increased metabolic demands and reduced developmental plasticity, we might have expected reduced *Ae. albopictus* performance in the FS condition across all competition treatments. It is possible that *Cx. pipiens,* which almost exclusively filter-feeds within the water column, forces *Ae. albopictus* to spend even more feeding time browsing surfaces than normal, which reduces *Ae. albopictus’* performance within highly degraded tires through a form of interference competition, which is defined as when the presence of one species alters the behavior of another [89]. Although past studies suggest that mosquitoes may be affected by interference competition produced by water-borne substances or aggression [89,90,91,92], competition between *Ae. albopictus* and *Cx. pipiens* is widely assumed to occur via resource depletion. Our study suggests that differential responses to tire contaminants and interference competition may also play an important role in the outcome of competition between these species.

Our study suggests that a species’ response to competition across varying environmental conditions is best estimated by combining survival with other demographic data on development time and body size in a composite index of population performance, such as *λ*′. This finding is consistent with that from Villena, Terry, Iwata, Landa, LaDeau and Leisnham [31], which showed that increasing female mass with increasing tire leachate concentration of *Ae. albopictus* offset concomitant decreases in survival, resulting in no changes in *λ*′. An important limitation of most past research that have studied the effects of tire leachate on aquatic organisms is that findings on any toxicological effects are limited to single parameters of fitness, usually that of survival, precluding inferences on populations over multiple generations. Addressing individual parameters of adult fitness, especially of adult body size, is also important when considering vector mosquitoes. Larger field-collected *Ae. aegypti* has been shown to have a greater frequency of dengue infection in Brazil, presumably because they have greater longevity and biting rates that offset decreases in viral susceptibility [93]. In this study, we observed increases in female body size of *Cx. pipens* in tires with greater tire leachate concentrations from higher exposure to UV-B radiation. *Cx. pipiens* is the main WNV vector of several avian species that serve to amplify the virus in urban areas [60]. *Cx. pipiens* also bite humans and plays a significant role in bridging WNV into human populations [60,62]. Our findings suggest that tires degraded by UV-B radiation might increase risks of human WNV transmission after *Ae. albopictus* invasion through two, interrelated mechanisms: by helping foster the area-wide persistence of *Cx. pipiens* and by increasing the probability that surviving *Cx. pipiens* adult females become infectious.

Our prediction regarding UV-B radiation impacts on tires was clearly supported. Dissolved and total zinc concentrations, which are common markers of tire leachate, were higher in tires exposed to UV-B radiation mimicking full sun conditions compared no UV-B conditions, while tires exposed to UV-B radiation mimicking shaded conditions exhibited intermediate zinc concentrations. These results are consistent with the only other study that has studied tire degradation among mosquito habitats in the field. Villena, Terry, Iwata, Landa, LaDeau and Leisnham [31] observed higher dissolved zinc concentrations from tires sampled in tire dumps, that were likely exposed to continuous full-sun conditions compared to those in auto-repair shops that probably experienced full or partial shade. Mean soluble zinc concentrations recorded by Villena, Terry, Iwata, Landa, LaDeau and Leisnham [31] at one dump site were 2.39 ± 1.17 (range: 0.05–7.26 mg/L), which was similar to the highest mean values of dissolved zinc in tires exposed to full sun conditions in our study. We based our UV-B radiation levels on measured values from the field. Although the levels of UV-B radiation exposure are likely to be highly variable among sites and temporally dynamic, our results suggest that our experimental conditions in the greenhouse were realistic to those in the field. This is one of the first studies to explore the decay of whole tires under realistic UV-B radiation conditions of terrestrial habitats. Most studies have used crumb rubber particles or tire chips to obtain tire leachate and assess its toxicity on organisms [27,30,31], or studied the effects of tires that are submerged in water [18,94,95].

Water-filled tire casings are often the most important development habitat for container utilizing mosquitoes worldwide due to their relatively large size and persistence in the environment [96,97]. Over 30 mosquito species, including seven invasive species, have been documented to use tire habitats in the United States [96]. The used tire industry has been influential in the spread of several species and their associated arthropod-borne viruses to new geographic areas in the United States and worldwide [98]. Several studies have documented the persistence of individual tires in the field for decades, where they provide habitat for successive generations of mosquitoes and are exposed to a range of adverse conditions that degrade their rubber and facilitate the leaching of compounds [99,100,101]. In this study, we focused on tire degradation from UV-B radiation because it is likely the most common source of tire degradation. Still, tires can also degrade as result of several other environmental processes, including temperature, water exposure, and other contaminants, such as road salt. Our findings that degraded tires with higher tire leachate promote condition-specific competition by reducing the competitive advantage of invasive *Ae. albopictus* over resident *Cx. pipiens* may apply to other mosquito communities and have wide-ranging impacts on disease transmission worldwide.

## Figures and Tables

**Table 1:** ANOVA results of the effects of UV-B radiation condition and competition treatment on estimated finite rate of population increase (λ’), survival, female developmental time, and female wing length of Ae. albopictus and Cx. pipiens. Ae. albopictus λ’ was log10(y+1) transformed. Significant (*p* < 0.05) results are bolded.

Variable	*λ’*			Survival			Female Developmental Time			Female Wing Length		
	df	F	*p*-Value	df	F	*p*-Value	df	F	*p*-Value	df	F	*p*-Value
*Ae. albopictus*												
UV-B condition	2,24	2.68	0.089	2,24	7.29	0.003	2,24	2.83	0.079	2,24	0.24	0.790
Competition	2,24	3.04	0.066	2,24	8.29	0.001	2,24	1.02	0.375	2,24	1.45	0.255
UV-B condition x competition	4,24	3.10	0.034	4,24	3.30	0.027	4,24	1.74	0.175	4,24	0.41	0.800
*Cx. pipiens*												
UV-B condition	2,24	2.97	0.066	2,24	5.91	0.008	2,21	0.37	0.692	2,21	13.7	0.002
Competition	2,24	0.15	0.752	2,24	3.79	0.037	2,21	5.38	0.013	2,21	6.11	0.008
UV-B condition x competition	4,24	0.01	0.990	4,24	0.28	0.888	4,21	1.13	0.370	4,21	2.61	0.064

## Data Availability

The data presented in this study are available in Dataset S1: Tire condition, Dataset S2: Competition trial: Dataset S3: Biofilm, Dataset S4: Mosquito zinc, Dataset S5: *Aedes* oviposition choice trail, Dataset S6: *Culex* oviposition choice trail.

## Supplementary Materials

The following are available online at https://www.mdpi.com/article/10.3390/insects13110969/s1, Figure S1: Ae. albopictus: UV-B radiation conditions and competition treatments; Figure S2: Cx. pipiens: UV-B radiation conditions and competition treatments; Dataset S1: Tire condition; Dataset S2: Competition trial; Dataset S3: Biofilm; Dataset S4: Mosquito zinc; Dataset S5: *Aedes* oviposition choice trial; Dataset S6: *Culex* oviposition choice trial.

## References

[1] Kesavaraju B., Juliano S.A. (2009). No evolutionary response to four generations of laboratory selection on antipredator behavior of *Aedes albopictus*: Potential implications for biotic resistance to invasion. J. Med. Entomol..

[2] Davis M.A. (2009). Invasion Biology.

[3] Lockwood J.L., Cassey P., Blackburn T.M. (2009). The more you introduce the more you get: The role of colonization pressure and propagule pressure in invasion ecology. Divers. Distrib..

[4] Tilman D. (1982). Resource Competition and Community Structure.

[5] Chase J.M., Leibold M.A. (2003). Ecological Niches: Linking Classical and Contempory Approaches.

[6] Case T. (1991). Invasion resistance, species build-up and community collapse in metapopulation models with interspecies competition. Biol. J. Linn. Soc..

[7] Lounibos L.P., Bargielowski I., Carrasquilla M.C., Nishimura N. (2016). Coexistance of *Aedes aegypti* and *Aedes albopictus* (Diptera: Culicidae) in peninsular Florida two decades after competitive displacements. J. Med. Entomol..

[8] Blaustein L., Chase J.M. (2007). Interactions between mosquito larvae and species that share the same trophic level. Annu. Rev. Entomol..

[9] Costanzo K.S., Kesavaraju B., Juliano S.A. (2005). Condition specific competition in container mosquitoes: The role of non-competing life-history stages. Ecology.

[10] Leisnham P.T., LaDeau S.L., Juliano S.A. (2014). Spatial and temporal habitat segregation of mosquitoes in urban Florida. PLoS ONE.

[11] Chesson P. (2000). Mechanisms of maintenance of species diversity. Annu. Rev. Ecol. Syst..

[12] Chesson P., Huntly N. (1997). The roles of harsh and fluctuating conditions in the dynamics of ecological communities. Am. Nat..

[13] Lawton J.H., Hassell M.P. (1981). Asymmetrical competition in insects. Nature.

[14] Juliano S.A. (2009). Species interactions among larval mosquitoes: Context dependence across habitat gradients. Annu. Rev. Entomol..

[15] Costanzo K.S., Muturi E.J., Lampman H.L., Alto B.W. (2011). The effects of resource type and ratio on competition with *Aedes albopictus* and *Culex pipiens* (Diptera: Culicidae). J. Med. Entomol..

[16] USTMA (2019). U.S. Scrap Tire Management Summary.

[17] Councell T.B., Dukenfield K.U., Landa E.R., Callender E. (2004). Tire-wear particles as a source of zinc to the environment. Environ. Sci. Technol..

[18] Day K.E., Holtze K.E., Metcalfe-Smith J.L., Bishop C.T., Dutka B.J. (1993). Toxicity of leachate from automobile tires to aquatic biota. Chemosphere.

[19] Selbes M., Yilmaz O., Khan A.A., Karanfil T. (2015). Leaching of DOC, DN, and inorganic constituents from scrap tires. Chemosphere.

[20] Capolupo M., Sørensen L., Jayasena K.D.R., Booth A.M., Fabbri E. (2020). Chemical composition and ecotoxicity of plastic and car tire rubber leachates to aquatic organisms. Water Res..

[21] Andrady A.L., Hamid H.S., Torikai A. (2003). Effects of climate change and UV-B on materials. Photochem. Photobiol. Sci..

[22] Andrady A.L., Hamid S.H., Hu X., Torikai A. (1998). Effects of increased solar ultraviolet radiation on materials. J. Photochem. Photobiol. Sci..

[23] Hartwell S.I., Jordahl D.M., Dawson C.E.O. (2000). The effect of salinity on tire leachate toxicity. Water Air Soil Pollut..

[24] Gualtieri M., Andrioletti M., Mantecca P., Vismara C., Camatini M. (2005). Impact of tire debris on in vitro and in vivo systems. Part. Fibre Toxicol..

[25] Gualtieri M., Andrioletti M., Vismara C., Milani M., Camatini M. (2005). Toxicity of tire debris leachates. Environ. Int..

[26] Crampton M., Ryan A., Eckert C., Baker K.H., Herson D.S. (2014). Effects of leachate from crumb rubber and zinc in green roofs on the survival, growth, and resistance characteristics of *Salmonella enterica* subsp. enterica serovar Typhimurium. Appl. Environ. Microbiol..

[27] Wik A., Dave G. (2005). Environmental labeling of car tires–toxicity to *Daphnia magna* can be used as a screening method. Chemosphere.

[28] Wik A., Dave G. (2006). Acute toxicity of leachates of tire wear material to *Daphnia magna*–variability and toxic components. Chemosphere.

[29] Wik A., Dave G. (2009). Occurrence and effects of tire wear particles in the environment–a critical review and an initial risk assessment. Environ. Pollut..

[30] Marwood C., McAtee B., Kreider M., Ogle R.S., Finley B., Sweet L., Panko J. (2011). Acute aquatic toxicity of tire and road wear particles to alga, daphnid, and fish. Ecotoxicology.

[31] Villena O.C., Terry I., Iwata K., Landa E.R., LaDeau S.L., Leisnham P.T. (2017). Effects of tire leachate on the invasive mosquito *Aedes albopictus* and the native congener *Aedes triseriatus*. PeerJ.

[32] Villena O.C., Momen B., Sullivan J., Leisnham P.T. (2018). Effects of ultraviolet radiation on metabolic rate and fitness of *Aedes albopictus* and *Culex pipiens* mosquitoes. PeerJ.

[33] Sanchez-Bayo F., Van den Brink P., Mann R. (2011). Ecological Impacts of Toxic Chemicals.

[34] Lefcort H., Hancock K.A., Maur K.M., Rostal D.C. (1997). The effects of used motor oil, silt, and the water mold *Saprolegnia parasitica* on the growth and survival of mole salamanders (genus *Ambystoma*). Arch. Environ. Contam. Toxicol..

[35] Lefcort H., Wehner E.A., Cocco P.L. (2013). Pre-exposure to heavy metal pollution and the odor of predation decrease the ability of snails to avoid stressors. Arch. Environ. Contam. Toxicol..

[36] Pollino C.A., Georgiades E., Holdway D.A. (2009). Physiological changes in reproductively active rainbowfish (*Melanotaenia fluviatilis*) following exposure to naphthalene. Ecotox. Environ. Safe..

[37] Aguirre A.A., Lutz P. (2004). Marine turtles as sentinels of ecosystem health: Is fibropapillomatosis an indicator?. EcoHealth.

[38] Zychowski G.V., Godard-Codding C.A.J. (2017). Reptilian exposure to polycyclic aromatic hydrocarbons and associated effects. Environ. Toxicol. Chem..

[39] Neff J.M., Stout S.A., Gunster D.G. (2005). Ecological risk assessment of polycyclic aromatic hydrocarbons in sediments: Identifying sources and ecological hazard. Integr. Environ. Assess. Manag..

[40] Honda M., Suzuki N. (2020). Toxicities of polycyclic aromatic hydrocarbons for aquatic animals. Int. J. Environ. Res. Public Health.

[41] Girardin V., Grung M., Meland S. (2020). Polycyclic aromatic hydrocarbons: Bioaccumulation in dragonfly nymphs (Anisoptera), and determination of alkylated forms in sediment for an improved environmental assessment. Sci. Rep..

[42] Rohr J., Crumrine P. (2005). Effects of a herbicide and an insecticide on pond community structure and processes. Ecol. Appl..

[43] De Hoop L., De Troch M., Hendriks A.J., De Laender F. (2013). Modeling toxic stress by atrazine in a marine consumer-resource system. Environ. Toxicol. Chem..

[44] Yee D.A., Kneitel J.M., Juliano S.A. (2010). Environmental correlates of abundances of mosquito species and stages in discarded vehicle tires. J. Med. Entomol..

[45] Lounibos L.P. (2002). Invasions by insect vectors of human disease. Annu. Rev. Entomol..

[46] Benedict M.Q., Levine R.S., Hawley W.A., Lounibos L.P. (2007). Spread of the tiger: Global risk of invasion by the mosquito *Aedes albopictus*. Vector-Borne Zoonotic Dis..

[47] Sprenger D., Wuithiranyagool T. (1986). The discovery and distribution of *Aedes albopictus* in Harris County, Texas. J. Am. Mosq. Control Assoc..

[48] Vinogradova E.B. (2000). Culex Pipiens Pipiens Mosquitoes: Taxonomy, Distribution, Ecology, Physiology, Genetics, Applied Importance and Control.

[49] Costanzo K.S., Mormann K., Juliano S.A. (2005). Asymmetrical competition and patterns of abundance of *Aedes albopictus* and *Culex pipiens* (Diptera: Culicidae). J. Med. Entomol..

[50] Leisnham P.T., LaDeau S.L., Saunders M.E.M., Villena O.C. (2021). Condition-specific competitive effects of the invasive mosquito *Aedes albopictus* on the resident *Culex pipiens* among different urban container habitats may explain their coexistence in the field. Insects.

[51] Carrieri M., Bacchi M., Bellini R., Maini S. (2003). On the competition occurring between *Aedes albopictus* and *Culex pipiens* (Diptera: Culicidae) in Italy. Environ. Entomol..

[52] Marini G., Guzzetta G., Baldacchino F., Arnoldi D., Montarsi F., Capelli G. (2017). The effect of interspecific competition on the temporal dynamics of *Aedes albopictus* and *Culex pipiens*. Parasites Vectors.

[53] Müller R., Knautz T., Vollroth S., Berger R., Kreb A., Reuss F., Groneberg D.A., Kuch U. (2018). Larval superiority of *Culex pipiens* to *Aedes albopictus* in a replacement series experiment: Prospects for coexistence in Germany. Parasites Vectors.

[54] Dehghan H., Sadraei J., Moosa-Kazemi S. (2010). The morphological variations of *Culex pipiens* larvae (Diptera: Culicidae) in Yazd Province, Central Iran. Iran. J. Arthropod-Borne Dis..

[55] Allgood D.W., Yee D.A. (2017). Oviposition preference and offspring performance in container breeding mosquitoes: Evaluating the effects of organic compounds and laboratory colonisation. Ecol. Entomol..

[56] Merritt R.W., Dadd R.H., Walker E.D. (1992). Feeding behavior, natural food, and nutritional relationships of larval mosquitoes. Annu. Rev. Entomol..

[57] Alto B.W., Lampman R.L., Kesavaraju B., Muturi E.J. (2013). Pesticide-induced release from competition among competing *Aedes aegypti* and *Aedes albopictus* (Diptera: Culicidae). J. Med. Entomol..

[58] Gerhardt R.R., Gottfried K.L., Apperson C.S., Davis B.S., Erwin P.C., Smith A.B., Panella N.A., Powell E.E., Nasci R.S. (2001). The first isolation of La Crosse virus from naturally occurring infected *Aedes albopictus*. Emerg. Infect. Dis..

[59] Kim C.H., Lampman R., Muturi E.J. (2005). Bacterial communities and midgut microbiota associated with mosquito populations from waste tires in east-central Illinois. J. Med. Entomol..

[60] Farajollahi A., Fonseca D.M., Kramer L.D., Kilpatrick M.A. (2011). “Bird biting” mosquitoes and human disease: A review of the role of *Culex pipiens* complex mosquitoes in epidemiology. Infect. Genet. Evol..

[61] Chancey C., Grinev A., Volkova E., Rios M. (2015). The global ecology and epidemiology of West Nile virus. BioMed Res. Int..

[62] Kilpatrick A.M., Meola M.A., Moudy R.M., Kramer L.D. (2008). Temperature, viral genetics, and the transmission of West Nile virus by *Culex pipiens* mosquitoes. PLOS Pathog..

[63] Tiawsirisup S., Platt K.B., Evans R.B., Rowley W.A. (2005). A comparision of West Nile virus transmission by *Ochlerotatus trivittatus* (COQ.), *Culex pipiens* (L.), and *Aedes albopictus* (Skuse). Vector-Borne Zoonotic Dis..

[64] Rizzoli A., Bolzoni L., Chadwick E.A., Capelli G., Montarsi F., Grisenti M., de la Puente J.M., Muñoz J., Figuerola J., Soriguer R. (2015). Understanding West Nile virus ecology in Europe: *Culex pipiens* host feeding preference in a hotspot of virus emergence. Parasites Vectors.

[65] Grant R.H., Apostol K.G., Schmitz H.F., Gao W., Slusser J.R., Schmoldt D.L. (2010). Physiological Impacts of Short-Term UV Irradiance Exposures on Cultivars of Glycine Max. UV Radiation in Global Climate Change: Measurements, Modeling and Effects on Ecosystems.

[66] Sullivan J.H., Pope L.C., Sutherland B.M., Bennett P.V., Blum J.E., Stapleton A.E., Gitz D.C., Gao W., Slusser J.R., Schmoldt D.L. (2010). Assessment of DNA Damage as a Tool to Measure UV-B Tolerance in Soybean Lines Differing in Foliar Flavonoid Composition. UV Radiation in Global Climate Change: Measurements, Modeling and Effects on Ecosystems.

[67] Maciá A. (2006). Differences in performance of *Aedes aegypti* larvae raised at different densities in tires and ovitraps under field conditions in Argentina. J. Vector Ecol..

[68] USEPA (2007). SW-846 Test Method 3015A: Microwave Assisted Acid Digestion of Aqueous Samples and Extracts.

[69] USEPA (1994). Method 200.7, Revision 4.4: Determination of metals and trace elements in water and wastes by inductively coupled plasma-atomic emission spectrometry. Methods for the Determination of Metals in Environmental Samples, Supplement I.

[70] Freed T.Z., Leisnham P.T. (2014). Roles of spatial partitioning, competition, and predation in the North American invasion of an exotic mosquito. Oecologia.

[71] Freed T.Z., Kesavaraju B., Leisnham P.T. (2014). Effects of competition and predation by native mosquitoes on the North American invasion of *Aedes japonicus japonicus* (Diptera: Culicidae). J. Med. Entomol..

[72] Griswold M.W., Lounibos L.P. (2005). Competitive outcomes of aquatic container Diptera depend on predation and resource levels. Ann. Entomol. Soc. Am..

[73] Juliano S.A. (1998). Species introduction and replacement among mosquitoes: Interspecific resource competition or apparent competition?. Ecology.

[74] Vinogradova E., Karpova S. (2006). Effect of photoperiod and temperature on the autogeny rate, fecundity and wing length in the urban mosquito, *Culex pipiens pipiens f. molestus* (Diptera, Culicidae). Int. J. Dipterol. Res..

[75] Lounibos L., Suárez S., Menéndez Z., Nishimura N., Escher R., Connell S.O., Rey J. (2002). Does temperature affect the outcome of larval competition between *Aedes aegypti* and *Aedes albopictus*?. J. Vector Ecol..

[76] Leisnham P.T., Scott B., Baldwin A.H., LaDeau S.L. (2019). Effects of detritus on the mosquito *Culex pipiens*: Phragmites and Schedonorus (Festuca) invasion affect population performance. Int. J. Environ. Res. Public Health.

[77] Zhang H., Liu J., Li C., Momen B., Kohanski R., Pick L. (2009). Deletion of *Drosophila* insulin-like peptides causes growth defects and metabolic abnormalities. Proc. Natl. Acad. Sci. USA.

[78] Braissant O., Wirz D., Gopfert B., Daniels A.U. (2010). Use of isothermal microcalorimeter to monitor microbial activities. FEMS Microbiol. Lett..

[79] Yee D.A., Glasgow W.C., Ezeakacha N.F. (2020). Quantifying species traits related to oviposition behavior and offspring survival in two important disease vectors. PLoS ONE.

[80] Allgood D.W. (2011). Influence of Detritus Levels and Organic Pollution on Interspecific Resource Competition, Oviposition Behavior, and Larval Survival of Two Tire-Inhabiting Mosquito Species (Diptera: Culicidae).

[81] SAS Institute (2003). SAS User’s Guide: Statistics, Version 9.1..

[82] Scheiner S.M., Scheiner S.M., Gurevitch J. (2001). MANOVA: Multiple Response Variables and Multispecies Interactions. Design and Analysis of Ecological Experiments.

[83] Dayton P., Dayton P.K. (1971). Competition, disturbance, and community organization: The provision and subsequent utilization of space in a rocky intertidal community. Ecol. Monogr..

[84] Juliano S.A. (2002). Invasion ecology of Asian tiger mosquito: Egg mortality alters the outcome of competition among larvae. Ecol. Soc. Am. Annu. Meet. Abstr..

[85] Malenke J.R., Newbold N., Clayton D.H. (2011). Condition-specific competition governs the geographic distribution and diversity of ectoparasites. Am. Nat..

[86] Aboelkheir M.G., Lima Junior J.G., Toledo Filho R.D., Souza Junior F.G., dos Santos Siqueira C.Y. (2021). Thermo-oxidative degradation of vulcanized SBR: A comparison between ultraviolet (UV) and microwave as recovery techniques. J. Polym. Res..

[87] Grech M.G., Juliano S.A. (2018). Complex effects of superior competitors and resources on *Culex restuans* (Diptera: Culicidae) oviposition. J. Med. Entomol..

[88] Nguyen T., Williams-Newkirk A., Kitron U., Chaves L. (2014). Seasonal weather, nutrients, and conspecific presence impacts on the southern house mosquito oviposition dynamics in combined sewage overflows. J. Med. Entomol..

[89] Silberbush A., Tsurim I., Rosen R., Margalith Y., Ovadia O. (2014). Species-specific non-physical interference competition among mosquito larvae. PLoS ONE.

[90] Broadie K.S., Bradshaw W.E. (1991). Mechanisms of interference competition in the western treehole mosquito, Aedes sierrensis. Ecol. Entomol..

[91] Sunahara T., Mogi M. (2002). Priority effects of bamboo-stump mosquito larvae: Influences of water exchange and leaf litter input. Ecol. Entomol..

[92] Talaga S., Dejean A., Mouza C., Dumont Y., Leroy C. (2018). Larval interference competition between the native Neotropical mosquito *Limatus durhamii* and the invasive *Aedes aegypti* improves the fitness of both species. Insect Sci..

[93] Juliano S.A., Ribeiro G.S., Maciel-De-Freitas R., Castro M.G., Codeco C., Lourenco-de-Oliveira R., Lounibos L.P. (2014). She’s a femme fatale: Low-density larval development produces good disease vectors. Mem. Do Inst. Oswaldo Cruz.

[94] Kellough R.M. (1991). The Effects of Scrap Automobile Tires in Water.

[95] Abernethy S.G., Montemayor B.P., Penders J.W. (1996). The Aquatic Toxicity of Scrap Automobile Tires.

[96] Yee D.A. (2008). Tires as habitats for mosquitoes: A review of studies within the eastern United States. J. Med. Entomol..

[97] LaDeau S.L., Leisnham P.T., Biehler D., Bodner D. (2013). Higher mosquito production in low-income neighborhoods of Baltimore and Washington, DC: Understanding ecological drivers and mosquito-borne disease risk in temperate Cities. Int. J. Environ. Res. Public Health.

[98] Bennett K.L., Gómez Martínez C., Almanza A., Rovira J.R., Mcmillan W.O., Enriquez V., Barraza E., Diaz M., Sanchez-Galan J.E., Whiteman A. (2019). High infestation of invasive *Aedes* mosquitoes in used tires along the local transport network of Panama. Parasites Vectors.

[99] Kwon E., Castaldi M.J. (2009). Fundamental understanding of the thermal degradation mechanisms of waste tires and their air pollutant generation in a N2 atmosphere. Environ. Sci. Technol..

[100] Kwon E.E., Castaldi M.J. (2012). Mechanistic understanding of polycyclic aromatic hydrocarbons (PAHs) from the thermal degradation of tires under various oxygen concentration atmospheres. Environ. Sci. Technol..

[101] Zheng S., Liao M., Chen Y., Brook M.A. (2020). Dissolving used rubber tires. Green Chem..

