# Supplementary figures and images for: The Role of Tire Leachate in Condition-Specific Competition and the Persistence of a Resident Mosquito from a Competitively Superior Invader
Source: Insects. 2022 Oct 22;13(11):969. doi: 10.3390/insects13110969 (PMC9693511; doi:10.3390/insects13110969)

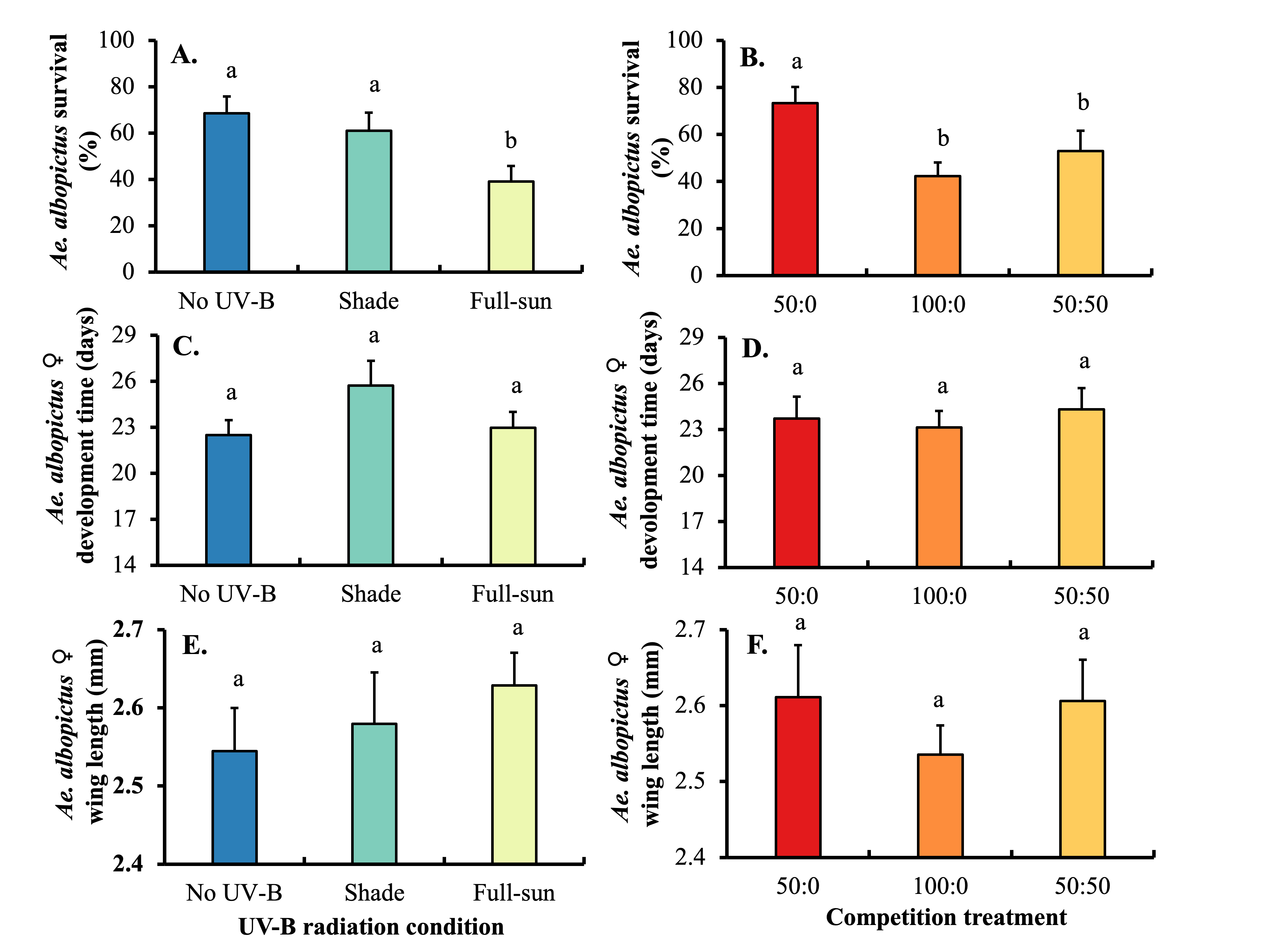

Supplement: Supplementary file 1 [file insects-13-00969-s001.zip › Figure S1.tif]

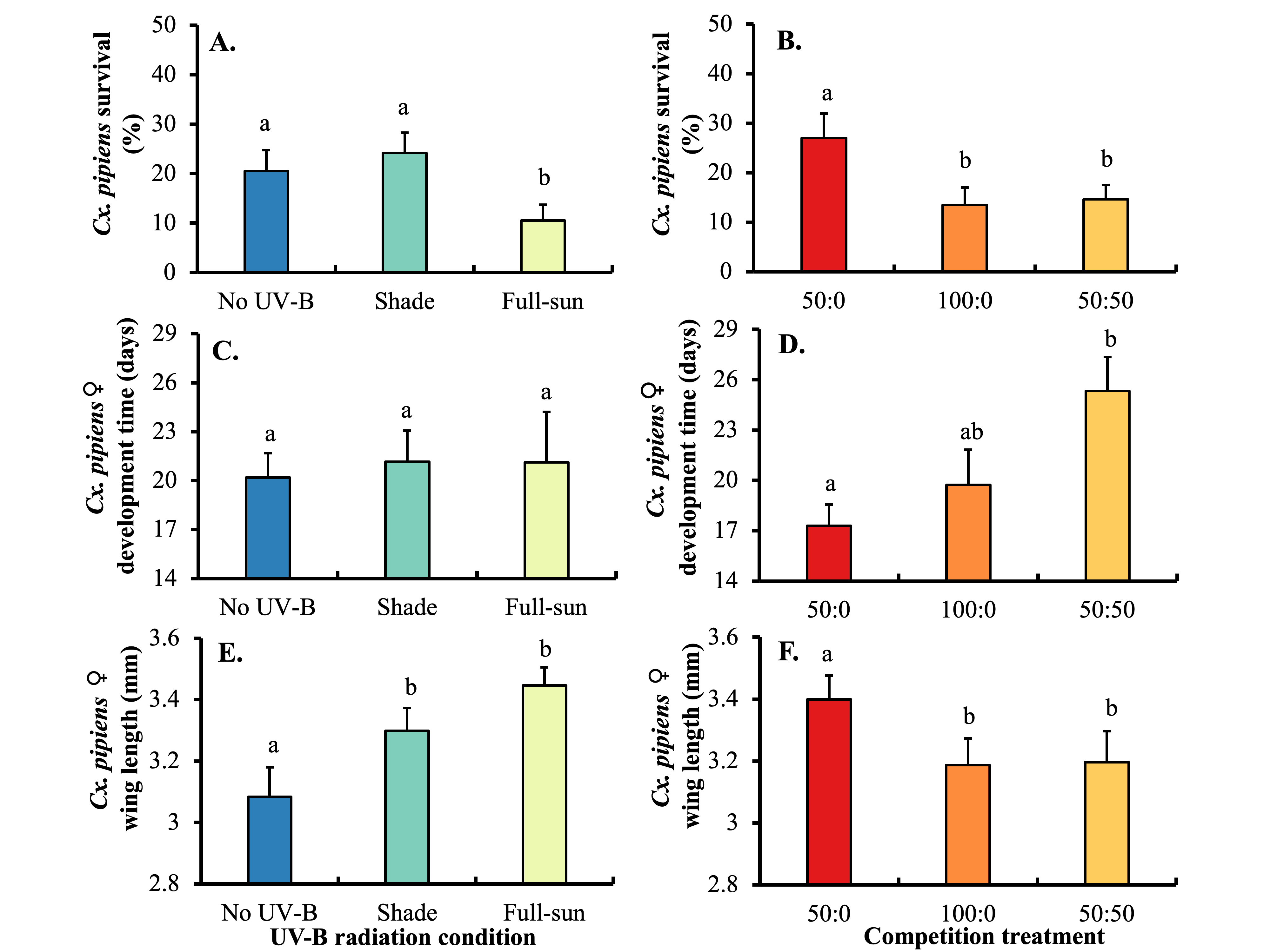

Supplement: Supplementary file 1 [file insects-13-00969-s001.zip › Figure S2.tif]
